# Adenovirus-mediated CD40L gene transfer increases Teffector/Tregulatory cell ratio and upregulates death receptors in metastatic melanoma patients

**DOI:** 10.1186/s12967-017-1182-z

**Published:** 2017-04-20

**Authors:** A. Schiza, J. Wenthe, S. Mangsbo, E. Eriksson, Anders Nilsson, T. H. Tötterman, A. Loskog, G. Ullenhag

**Affiliations:** 1 Department of Immunology, Genetics and Pathology, Uppsala University, Uppsala, Sweden; 2 Department of Oncology, Uppsala University Hospital, Entrance 78, 751 85 Uppsala, Sweden; 3 Division of Radiology, Uppsala University Hospital, Uppsala, Sweden

**Keywords:** AdCD40L, Malignant melanoma, Immunotherapy, Proteomics, T regulatory cells, Myeloid-derived suppressor cells

## Abstract

**Background and aims:**

Malignant melanoma is an aggressive tumor sensitive for immunotherapy such as checkpoint blockade antibodies. Still, most patients with late stage disease do not respond, and the side effects can be severe. Stimulation of the CD40 pathway to initiate anti-tumor immunity is a promising alternative. Herein, we demonstrate immune profiling data from melanoma patients treated with an adenovirus-based CD40 ligand gene therapy (AdCD40L).

**Methods:**

Peripheral blood mononuclear cells and plasma were collected from malignant melanoma patients (n = 15) enrolled in a phase I/IIa study investigating intratumoral delivery of AdCD40L with or without low dose cyclophosphamide. Cells were analyzed by flow cytometry while plasma samples were analyzed by a multi-array proteomics.

**Results:**

All patients had an increased Teffector/Tregulatory cell ratio post therapy. Simultaneously, the death receptors TNFR1 and TRAIL-R2 were significantly up-regulated post treatment. Stem cell factor (SCF), E-selectin, and CD6 correlated to enhanced overall survival while a high level of granulocytic myeloid-derived suppressor cells (gMDSCs), IL8, IL10, TGFb1, CCL4, PlGF and Fl3t ligand was highest in patients with short survival.

**Conclusions:**

AdCD40L intratumoral injection induced desirable systemic immune effects that correlated to prolonged survival. Further studies using CD40 stimulation in malignant melanoma are warranted.

*Trial registration* The 002:CD40L trial “Phase I/IIa AdCD40L Immunogene Therapy for Malignant Melanoma and Other Solid Tumors” (clinicalTrials.gov identifier: NCT01455259) was registered at September 2011

## Background

Malignant melanoma (MM) is considered sensitive to immunotherapy due to the immunogenic nature of the tumor [1]. Understanding the interactions between the cells of the immune system and the tumor is the cornerstone for the development of new anticancer treatments. Tumor cells avoid destruction by T cells and natural killer (NK) cells by releasing immunosuppressive substances promoting accumulation and/or differentiation of different types of suppressive cells. These suppressive cells are derived from myeloid and lymphocytic progenitors or even mature immune phenotypes such as naive CD4+ lymphocytes. Collectively, these are commonly referred to as myeloid-derived suppressor cells (MDSCs) and regulatory T cells (Tregs). It has been shown that elevated levels of both MDSCs and Tregs are positively correlated with tumor burden [2, 3]. MDSCs are a heterogeneous population of cells that potently suppress various T cell functions and can participate to induce, or recruit, Tregs [4]. Tregs can modulate the immune system by secreting the inhibitory cytokines interleukin 10 (IL-10) and transforming growth factor beta (TGF-β), by inducing apoptosis of effector lymphocytes directly or indirectly by depriving T effector cells (Teff) of cytokines. They also have the capacity to inhibit dendritic- and NK cell functions [5].

Immune therapy for various types of cancer is based on the principle that the patient’s immune system is capable of generating immune responses against tumor cells [6]. Decades of basic research about how the immune system and tumor cells interact has lead to important clinical achievements such as the development of the immunomodulatory antibodies ipilimumab (anti-CTLA-4) and subsequently the more efficient pembrolizumab and nivolumab (PD-1 inhibitors). However, side effects of these new therapeutic agents are substantial and the majority of the patients with metastatic disease does not respond [7]. Hence, there is still a great need for other treatment options including additional combination treatment strategies.

AdCD40L is a replication-deficient adenovirus carrying the gene for human CD40 ligand (CD40L). CD40L stimulates myeloid cells such as dendritic cells (DCs) and macrophages via the CD40 receptor. This CD40L/CD40 interaction leads to the maturation of DCs, stimulates T helper 1(Th1)-type immune responses and promotes T cell activation and migration into the tumor microenvironment [8, 9]. We have previously reported clinical results from a phase I/IIa study in which AdCD40L was administered by ultrasound-guided intratumoral injections in patients conditioned with cyclophosphamide. In the study, AdCD40L could decrease tumor activity and responding patients commonly had higher levels of T cells than patients not responding to treatment [10]. In the current study, we sought to investigate the capacity of locally administered AdCD40L therapy to activate systemic immunity by flow cytometry and multi-array proteomics, and if such responses correlated to patient survival.

## Methods

### Trial design

Peripheral blood mononuclear cells (PBMCs) and plasma from patients enrolled in the 002:CD40L trial “Phase I/IIa AdCD40L Immunogene Therapy for Malignant Melanoma and Other Solid Tumors” (clinicalTrials.gov identifier: NCT01455259) was taken before treatment and at different time points after treatment initiation. The samples were frozen prior analysis. The study protocol and sampling procedures were approved by the Regional Ethical Committee and the Swedish Medical Products Agency. The trial design and results have been presented elsewhere [10]. Briefly, six patients were treated with intratumoral AdCD40L (2.5 × 10e11 VP) injections (cohort 1), four times, once weekly. Nine additional patients received low-dose intravenous cyclophosphamide (300 mg m^2^) conditioning 1–2 days before the first and fourth AdCD40L injection (cohort 2). Two patients in cohort 2 received another cycle of AdCD40L and cyclophosphamide. All patients received treatments in one selected lesion.

### Flow cytometry

PBMCs from pre, at week 5, and at week 9 after treatment initiation were analyzed by flow cytometry. The cells were thawed, washed with phosphate-buffered saline (PBS) and incubated with FcR blocking reagent (Miltenyi Biotech, Bergisch Gladbach, Germany) for 10 min. Cells were then stain with specific antibody or isotype control for 30 min at 4 °C before suspended in 0.5% bovine serum albumin (BSA) (Sigma-Aldrich, St. Louis, USA) in 1×PBS. Cells stained for FOXP3 were fixed and permeabilized with FOXP3 fix/perm buffer set from Biolegend (San Diego, CA, USA) according to manufacturer’s protocol. Antibodies used: αCD2 FITC (clone RPA.2.10), αCD56 FITC (clone HCD45), αCD11b PE/Cy7 (clone ICRF44), αCD14 APC/Cy7 (clone HCD14), αCD33 BV421 (clone WM53), αHLA DR 510 (clone L234), αCD3 FITC (clone UCHT-1), αCD16 PE/Cy7 (clone 3G8), αCD56 APC (clone HCD56), αCD3 FITC (clone UCHT-1), αCD4 PerCP (clone OKT4), αCD127 Brilliant Violet 421 (cloneA019D5), αFOXP3 Alexa Flour 649 (clone 206D), and mouse IgG1κ Alexa Flour 649 (clone MOPC-21), all purchased from Biolegend. Cells were analyzed in a BD FACS Canto II (BD Biosciences, San Jose, CA, USA) and data was evaluated in Flow Jo (Tree Star, Ashland, OR, USA). Before gating of specific cell subsets, dead cells and duplets were removed by gating.

T cells were determined as CD3 positive cells that either were of effector type (CD4−, CD127+) or regulatory type (CD4+FoxP3+CD127−). NK cells were CD3− negative cells expressing CD16 and CD56. Monocytic myeloid suppressors were CD14+CD11b+CD33+ cells that lacked HLA-DR while granulocytic myeloid suppressors were CD11b+CD33+ cells lacking HLA-DR and CD14.

### Plasma analyses

Plasma samples taken before treatment (pre) and at 3 weeks post treatment initiation (post) were analyzed using ProSeek Multiplex Inflammation by the company service facility (Olink Proteomics AB, Uppsala, Sweden). Proseek multiplex is an antibody-based method for multiplex protein detection. Two antibodies labeled with unique oligonucleotide sequences need to bind to the same protein for positive detection. Upon antibody proximity, the oligonucleotides will overlap and act as primers for a quantitative PCR-based detection. Hence, the method is highly specific and sensitive and allows multiplexing with low background [11]. The values are reported in relative units referred to as normalized protein expression (NPX) since the data generated cannot be converted to absolute concentrations.

### Statistical evaluations

All statistical analyses were performed using the Prism Software (Graphpad Software Inc., La Jolla, CA, USA). Correlation analyses were performed using the nonparametric Spearman rank correlation. Wilcoxon matched-pairs signed rank test was used to determine differences between pre and post treatment samples. For comparisons between unpaired groups, Mann–Whitney U testing was used since data distribution could not be assumed to have normal distribution. A p value <0.05 was considered significant.

## Results

### AdCD40L induces effector T cell responses

**Fig. 1:**
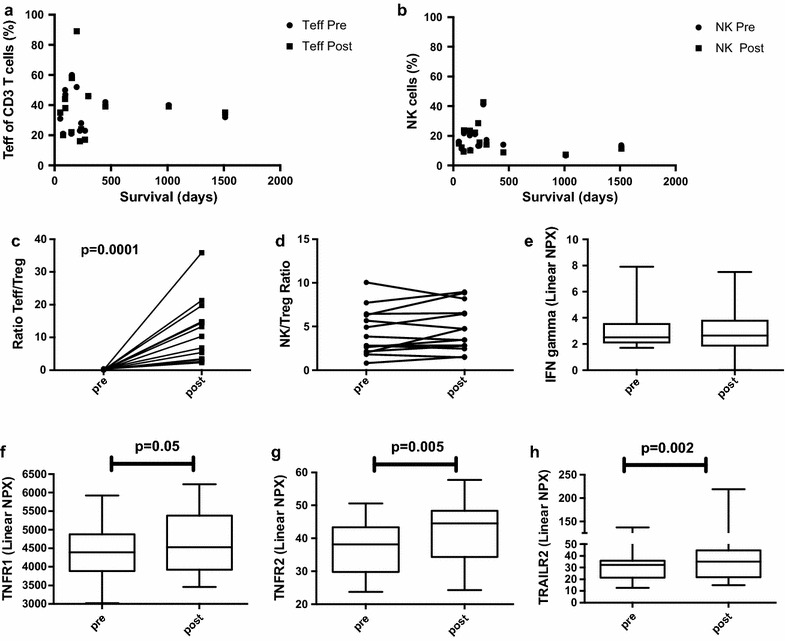
PBMCs were evaluated for the presence of T effector cells (CD3+CD4−CD127+), Tregulatory cells (CD3+CD4+FoxP3+CD127−) and NK cells (CD3−CD16+CD56+). **a**, **b** The percentage of T effector cells and NK cells before treatment (pre) and 3 weeks post treatment initiation (post) were displayed against survival. **c**, **d** The ratio of Teff/Treg and NK/Treg were calculated pre and post 3 weeks. **e**–**h** Patient plasma was analyzed by ProSeek proteomics at baseline (pre) and after 3 weeks post treatment initiation (post) and proteins connected to T cell activity are displayed. Statistical calculations were done by GraphPad Prism utilizing Wilcoxon test for paired samples

In the current paper, we performed immunoprofiling of patients participating in the 002:CD40L trial to define the mechanisms of action of AdCD40L and to find biomarkers that correlate with survival. PBMCs were purified from patient blood and the immune profile was investigated with flow cytometry. We have previously shown that the level of effector T cells did not change significantly over time. In this paper we show that neither T effector cells nor NK cells correlate with survival (Fig. 1a, b). However, all patients had an increase of the Teff/Treg ratio post therapy comparing the ratio pretreatment to that of 3 weeks post treatment initiation (p = 0.0001) (Fig. 1c), which was not evident for the NK/Treg ratio (Fig. 1d). However, this did not result in enhanced IFNg, as measured in plasma 3 weeks post treatment (Fig. 1e). Moreover, fold change of the analytes and of Tregs did not correlate. Nevertheless, death receptors used by T cells to induce tumor cell apoptosis were increased post treatment (Fig. 1f, TNFR1, p = 0.05; and 1H, TRAILR2, p = 0.002) as well as TNFR2, Fig. 1g; p = 0.005). Hence, AdCD40L seems an important inducer of T cell activation.

### Granulocytic MDSCs correlate with poor survival

**Fig. 2:**
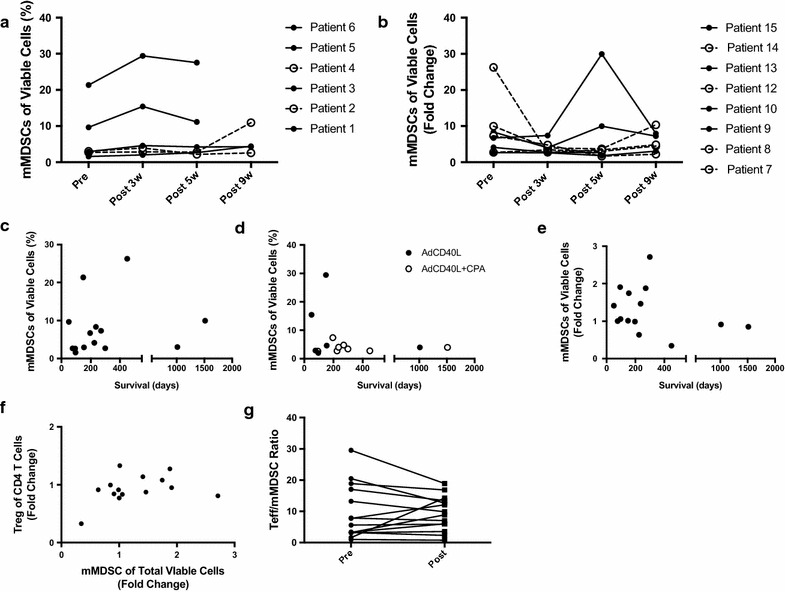
PBMCs were evaluated for the presence of monocytic MDSCs (CD11b+CD14+CD33+HLA-DR−) at different time points post treatment initiation (**a**, **b**) and correlated to overall survival (**c** Pre, **d** Post, **e** fold change) or Tregs (CD3+CD4+FoxP3+CD127−) (**f**) using Spearmans test for nonparametric samples. *Open circles* in **a** and **b** indicates responding patients, as determined by having decreased activity in tumor lesions as defined by PET/CT. **g** The ratio of Teff/mMDSC was calculated before and at 3 weeks post treatment initiation. Significant difference was calculated using Wilcoxon testing for paired samples. GraphPad Pris was used for statistical calculations

**Fig. 3:**
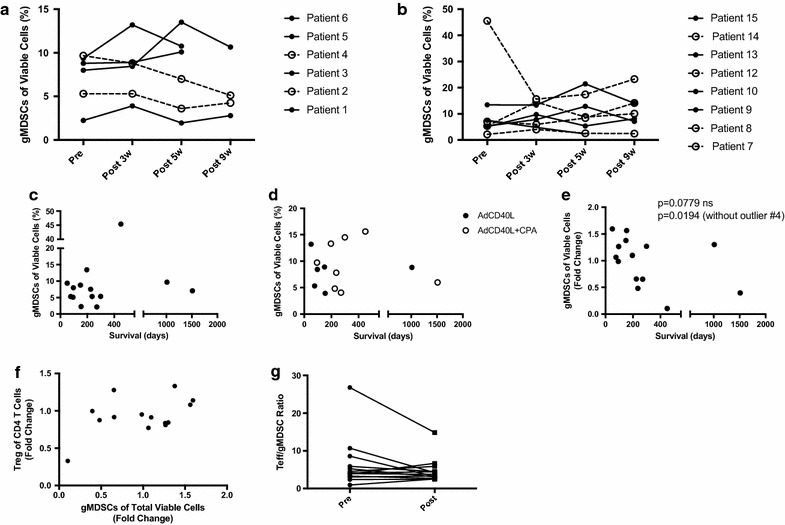
PBMCs were evaluated for the presence of granulocytic MDSCs (CD11b+CD14-CD33+HLA-DR−) at different time points post treatment initiation (**a**, **b**) and correlated to overall survival (**c** Pre, **d** Post, **e** fold change) or Tregs (CD3+CD4+FoxP3+CD127−) (**f**) using Spearmans test for nonparametric samples. *Open circles* in **a** and **b** indicates Responding patients, as determined by having decreased activity in tumor lesions as defined by PET/CT. **g** The ratio of Teff/gMDSC was calculated before and at 3 weeks post treatment initiation. Significant difference was calculated using Wilcoxon testing for paired samples. GraphPad Pris was used for statistical calculations

In animal models, AdCD40L reduces the presence of MDSCs in the tumor post treatment [12]. Hence, the level of suppressive myeloid cells was analyzed in patient participating in the 002:CD40L trial. The level of CD14+ (monocytic) myeloid suppressor cells (mMDSCs) was evaluated over time in patient blood. Responding patients, as determined by having decreased activity in tumor lesions as defined by PET [10], had in general low level of MDSCs compared to the other patients at 5 weeks post treatment (Fig. 2a, b). Nevertheless, mMDSC level before (Fig. 2c) or after three doses of AdCD40L (Fig. 2d, e) did not inversely correlate to overall survival (OS). Neither did the fold change pre versus post treatment of mMDSCs correlate with the corresponding Treg values (Fig. 2f). Most patients had a decreased Teff:mMDSC ratio post treatment but this did not reach significance (Fig. 2g). The CD14- (granulocytic) MDSCs (gMDSCs) were evaluated over time. Responding patients had at some point decreasing levels of gMDSCs (Fig. 3a, b) but the gMDSC level before (Fig. 3c) or after three doses of AdCD40L (Fig. 3d) did not inversely correlate with survival. Nevertheless, the level of gMDSCs varied among the patients. If fold change values were calculated it was clear that greater increases of gMDSCs pre and post therapy correlated inversely to survival. The analysis was conducted two times, one with the outlier and one without. Since the outlier alone, affected a significant association, the outlier was discarded as suggested by Karen Grace-Martin (http://www.theanalysisfactor.com) (Fig. 3e). However, fold change of gMDSCs did not correlate to the corresponding value of Tregs in these patients (Fig. 3f). Further, the Teff:gMDSC ratio pre and 3 weeks post treatment initiation was not significantly different (Fig. 3g).

### Factors determining poor survival post AdCD40L therapy

**Fig. 4:**
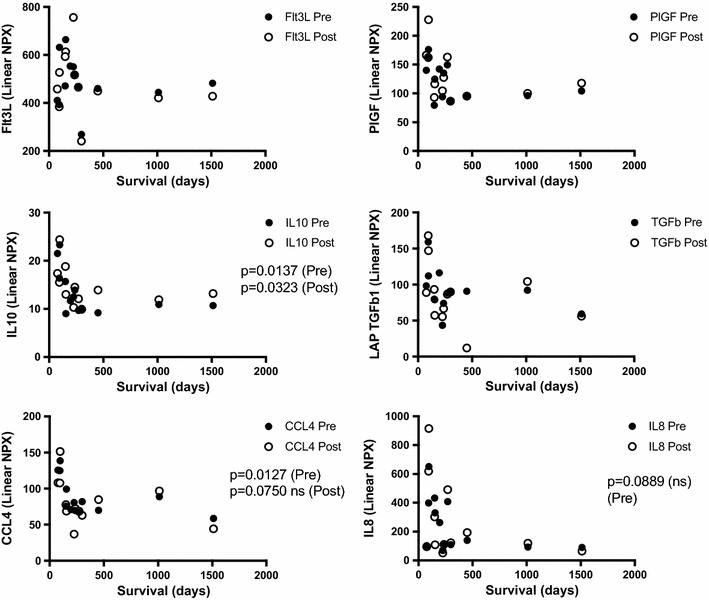
Patient plasma was analyzed by ProSeek proteomics at baseline (pre) and after 3 weeks post treatment initiation (post) and correlated to OS. Proteins connected to immunosuppression are displayed. Statistically significant correlations to OS were calculated using Spearmans test for nonparametric samples. GraphPad Prism was used for statistical calculations

**Fig. 5:**
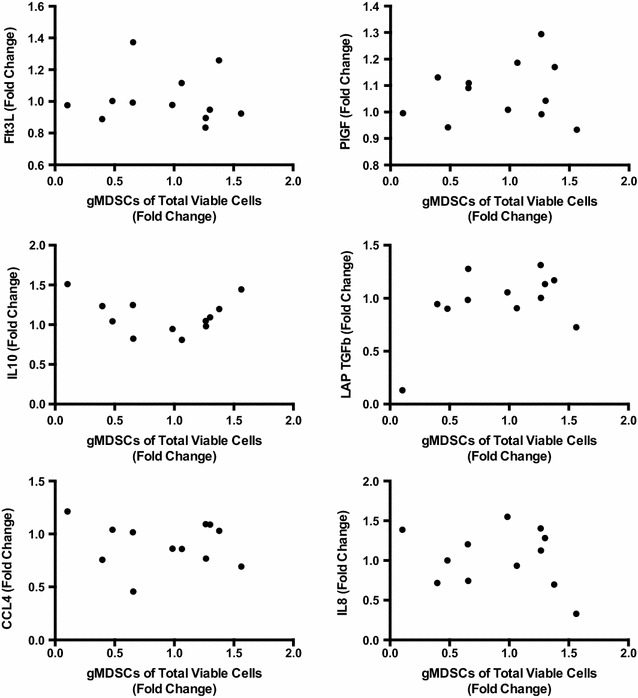
Patient plasma was analyzed by ProSeek proteomics at baseline (pre) and after 3 weeks post treatment initiation (post) and fold changes were calculated for these time points for each analyte. The fold change of analytes were then correlated to the fold change of gMDSCs (CD11b+CD14−CD33+HLA-DR−) pre and at 3 weeks post treatment initiation using Spearmans test for nonparametric samples. GraphPad Prism was used for statistical calculations

Next, soluble factors in patient blood were investigated by proteomics before treatment and at 3 weeks post treatment initiation. The analysis revealed that the most common factors of immunosuppression such as TGFb1 did not negatively correlate with survival (Fig. 4). However, the higher levels of TGFb1 but also of Fms-like tyrosine kinase 3 ligand (Flt3L) and interleukin-8 (IL-8) were observed among the patients with survival shorter than 6 months. Further, similar clusters were noted for placental growth factor (PlGF), IL10 and CCL4. In fact, IL10 had in inverse correlation to survival both in pre and post samples and the CCL4 level post treatment was inversely correlated to survival. Since, granulocytic MDSCs showed a correlation with poor survival, we investigated if any of these plasma proteins were correlated to the fold change of gMDSCs. However, there were no significant correlations between these markers and the presence of these cells (Fig. 5).

### CD6 and SCF correlate to overall survival

**Fig. 6:**
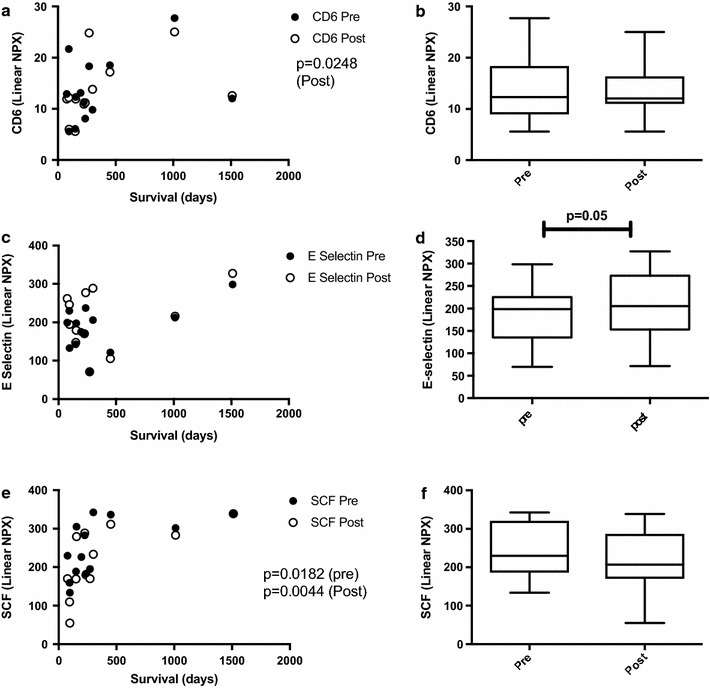
Patient plasma was analyzed by ProSeek proteomics at baseline (pre) and after 3 weeks post treatment initiation (post) and proteins connected to immunity are displayed in the graphs. Analytes were correlated to OS using Spearmans test for nonparametric samples (**a**, **c**, **e**) and significant difference pre and at 3 weeks post treatment initiation was calculated using Wilcoxon test for paired samples (**b**, **d**, **f**). GraphPad Prism was used for statistical calculations

AdCD40L is an immunostimulating therapy but we did not see any changes of common myeloid cell activating markers such as interleukin 12 (IL12) or TNF (data not shown). However, the post value of CD6, which is important in the formation of the immunological synapse, was correlated to patient OS (Fig. 6a; p = 0.0248). However, comparing the level of CD6 pre and post treatment did not reveal an increase of the plasma level per se (Fig. 6b). E-selectin that is important for T cell attachment and migration into the tumor lesion via the vascular system was higher in patients that survived long but it did not correlate with OS (Fig. 6c). Nevertheless, the plasma level was increased post therapy (Fig. 6d; p = 0.05). Interestingly, stem cell factor (SCF) correlated with OS both pre and post treatment (Fig. 6e; p = 0.0182 (pre) and p = 0.0044 (post) but the plasma levels were not increased post treatment (Fig. 6f).

## Discussion

We have previously conducted a phase I/IIa study in metastatic MM patients using the immune modulator AdCD40L [10]. In the current report, we have assessed whether local AdCD40L treatment could stimulate systemic immune activation and reduce the level of immunosuppressive cells and their suppressive mediators.

In patients with bladder cancer, local administration of AdCD40L immunogene therapy led to stimulation of Th1-type of immunity and suppression of Tregs [9]. Tregs are overrepresented in the blood of patients with metastatic MM [13–15] and an increase in the Treg level in tumors is generally a poor prognostic factor since Tregs suppress T effector cells hampering antitumor immune responses [16]. A shift in the ratio of Tregs:Teff in favor of Teff, suggests an increased efficacy of immunotherapy treatments [17]. Indeed, in our small cohort we observed that all the patients had an increased Teff/Treg ratio in blood 3 weeks post therapy. However, we did not analyze the specificity of these T cells and they could be targeting the virus as well as tumor antigens. No statistically significant difference was found between the patients in cohort 1 and in cohort 2, or between responding patients and the rest. Increased Teff/Treg ratio did not result in higher levels of the effector cytokine IFNg. However, IFNg is commonly acting locally in a cell-to-cell manner and is not easily detected systemically. TNF has both proinflammatory and immunosuppressive effects depending on the duration and state of the cancer disease; it can overcome the anergic state of T cell receptor-stimulated Tregs but a chronic exposure to TNF leads to an activation of Tregs [18–20]. TNF exerts its functions via two receptors, TNFR1 and TNFR2, with rather distinct roles in immune regulation. Action through TNFR1 leads to apoptotic pathways while action through TNFR2 is related to T cell survival. However, there is a degree of receptor crosstalk and overlapping function, depending on the activation state of the cell [21]. TNF-related apoptosis-inducing ligand (TRAIL) can preferentially induce apoptosis in malignant cells by its interaction with the death receptors; TRAIL-R1 and -R2 [22, 23]. In our material, we observed that patients with long survival and radiological response in general had higher plasma levels of TNFR1, and TRAILR2 with a few exceptions. Unexpectedly, patients #3 and #6 had the highest increase of the level of TNFR2 and they expressed the highest levels of Tregs in cohort 1. The same pattern is present in cohort 2; the patients with the higher increases in TNFR2 levels were those with the higher levels of Tregs post therapy.

In order to investigate if local AdCD40L therapy could reduce the number of systemic immunosuppressive cells, PBMCs were also evaluated for the presence of myeloid suppressor cells. MDSCs promote angiogenesis, tumor invasion and metastases through different soluble factors [4]. In humans, the two major subpopulations of MDSCs are often defined as mMDSCs and gMDSCs [24]. The number of MDSCs in the peripheral blood of MM patients correlated with clinical outcome [25]. We found neither a correlation between the level of MDSCs and OS, nor a correlation between the levels of MDSCs compared with Tregs. However, the fold increase of gMDSCs post therapy was greatest in the patients with the shortest survival. Furthermore, the most common suppressive molecules correlated neither to the level of MDSCs nor their fold change levels.

Plasma proteomics showed that some proteins, such as E-selectin, CD6 and stem cell factor (SCF) that are associated with immunity were altered post therapy. Adhesion molecules have an important role in cancer pathophysiology [26]. Selectins’ cardinal role, in the recruitment of leukocytes to sites of inflammation, is enhancement of leukocyte rolling. CD40/CD40L ligation induces E-selectin dependent attachment of T cells to the endothelium [27]. We found that the blood levels of vascular E-selectin were higher in patients with the longest OS and that they were significantly raised after treatment initiation. CD6 is a lymphocyte surface receptor involved in T cell development and activation as well as in the generation and/or function of several peripheral T cell subpopulations. It facilitates the adhesion between T cells and antigen-presenting cells (APCs) [28]. In our material, although no change in the level of CD6 in plasma post AdCD40L treatment was noticed, the post value of CD6 was found to be positively correlated to longer OS. C-KIT signaling is aberrantly activated in cancer and C-kit mutations are associated with MM [29]. Furthermore, loss of c-Kit is associated with melanoma progression [30]. SCF is the ligand for c-KIT and inhibits the growth of KIT-expressing melanoma cells [31]. In addition, SCF can dramatically enhance in vivo recovery of DCs [32]. In spite of the fact that the plasma levels of SCF were not affected by AdCD40L treatment, the patients with the longest survival had indeed higher SCF values post therapy.

Immunosuppressive factors were also altered post AdCD40L therapy. The PlGF, a member of the vascular endothelial growth factor (VEGF) family, enhances proliferation, migration and survival of cancer cells in both animal models and humans [33, 34]. Specifically, PlGF promotes melanoma aggressiveness [33]. We observed that the patients with survival shorter than 6 months had high levels of PlGF. TGF-b1 promotes angiogenesis, inhibition of NK cells and lymphocyte-activated killer cells and swift of Teff into Tregs [35]. IL-10 is a key immunosuppressive cytokine that impairs proliferation, cytokine production and capacity of Teff to migrate as well as targeting DCs [36]. In our material, TGFb1 was not significantly correlated with OS while both pre and post IL10 values correlated inversely to survival. Further, the patients with survival shorter than 6 months had high levels of IL10 and the majority of them also of TGFb1. IL-8 is a proinflammatory chemokine attracting neutrophils, monocytes as well as MDSCs [37]. IL-8 can also promote angiogenesis [37]. High plasma concentrations of IL-8 correlate with poor prognosis [38, 39] and we have previously reported that the patients with the best survival in the present study experienced a pronounced decrease of intratumoral IL8. This is in line with the finding that the patients with short survival had very high plasma levels of IL-8 in plasma post therapy while the patients with the best responses had considerably lower levels. Chemokine (C–C motif) ligand 4 (CCL4) is a chemokine attracting Tregs [40], which could explain the finding that the plasma levels of CCL4 post treatment, was inversely correlated to OS. Finally, Flt3L mobilizes hematopoietic progenitors from the bone marrow into the peripheral blood and secondary lymphatic organs [41]. A few animal studies have reported that the administration of Flt3L enhances Th1- immune responses and anti-tumor activity [42, 43]. However, other animal studies suggest that Flt3L activates the transcription factor STAT3 that stimulates the expansion and function of MDSCs [44, 45]. In our study, the level of Flt3L in plasma after treatment was higher among patients with shorter survival and in five of these patients, mMDSCs were indeed higher.

Comparisons of immune results between cohort 1 and 2 did not reveal any significant differences and the results are therefore not separated herein. We did not observe high levels of all cytokines in the same patient. Hence, an increased level of a certain cytokine compared to other patients in the study is not a reflection of a generally higher detection in that sample. Metastatic ocular melanoma is less immunogenic than metastatic cutaneous MM. It is refractory to all conventional therapy and trials with immunotherapeutic agents did not show promising results [46]. In an attempt to examine whether patients with metastatic ocular melanoma respond in a different way to immunotherapy compared to other types of metastatic MM, we created a subgroup consisting of these patients in our study (n = 8) that was compared to the rest. We were unable to detect any significant differences between these subgroups (data not shown). However, our findings indicate that it is not more difficult to evoke an immune response with AdCD40L in ocular melanoma patients, which is in line with the OS for individual patients that we previously have reported [10].

All patients enrolled in this last line study had a poor immunological capacity since they were heavily pre-treated. It cannot be excluded that blood collections in shorter intervals, e.g. in a weekly basis, could have revealed a different pattern in the proteomic profile. This is important to consider in future studies in order to early identify the best responders and thereby enabling the selection of subgroups for further treatment with AdCD40L.

## Conclusions

In conclusion, we report that the adenovirus-mediated CD40L gene transfer consistently increased the Teff/Treg ratio in the PBMCs while the fold induction of gMDSCs correlated inversely to survival. In addition, plasma proteomics revealed upregulation of molecules connected to T cell migration and interaction with DCs while immunosuppressive molecules were found high in patients with survival shorter than 6 months. The results imply that CD40 stimulation could be an effective approach for patients with MM. Further trials with larger patient cohorts and possible improvements of the treatment scheme are warranted.

## Abbreviations

APCs: antigen presenting cells
CCL4: chemokine (C–C motif) ligand 4
CD40L: CD40 ligand
DCs: dendritic cells
Flt3L: Fms-like tyrosine kinase 3 ligand
IFNg: interferon gamma
IL-8: interleukin-8
IL-10: interleukin 10
IL-12: interleukin 12
MDSCs: myeloid-derived suppressor cells
gMDSCs: granulocytic MDSCs
mMDSCs: monocytic MDSCs
MM: malignant melanoma
NK: natural killer
OS: overall survival
PBMCs: peripheral blood mononuclear cells
PlGF: placental growth factor
Teff: T effector cells
TGF-β: transforming growth factor beta
Th1: T helper 1
TRAIL: TNF-related apoptosis-inducing ligand
Tregs: regulatory T cells
SCF: stem cell factor
VEGF: vascular endothelial growth actor
